# Visual outcomes, spectacle independence, and patient satisfaction of pseudophakic mini-monovision using a new monofocal intraocular lens

**DOI:** 10.1038/s41598-022-26315-7

**Published:** 2022-12-15

**Authors:** Ella SeoYeon Park, Hyunmin Ahn, Sung Uk Han, Ikhyun Jun, Kyoung Yul Seo, Eung Kweon Kim, Tae-im Kim

**Affiliations:** 1 The Institute of Vision Research, Department of Ophthalmology, Severance Hospital, Yonsei University College of Medicine, 50-1 Yonsei-Ro, Seodaemungu, Seoul, 03722 Republic of Korea; 2 Corneal Dystrophy Research Institute, Department of Ophthalmology, Yonsei University College of Medicine, Seoul, Republic of Korea; 3 Saevit Eye Hospital, Goyang-Si, Gyeonggi-Do Republic of Korea

**Keywords:** Health care, Medical research

## Abstract

Modified monovision—or “mini-monovision”—is an alternative method to multifocal intraocular lenses (IOLs) for treating presbyopia. This study aimed to evaluate the clinical outcomes of patients bilaterally implanted with the new enhanced monofocal Tecnis Eyhance (ICB00) IOLs with the mini-monovision technique to improve near vision. In this retrospective case series, the medical records of 50 patients (100 eyes) who underwent bilateral cataract surgery were reviewed. Patients were divided into the Emmetropia and Mini-monovision groups based on the postoperative spherical equivalent and residual myopia. The binocular visual acuity for far (4 m), intermediate (66 cm), and near (40 cm) distances, binocular defocus curves, contrast sensitivity, visual symptoms, spectacle independence, and patient satisfaction rates were evaluated at 3 months postoperatively. The binocular uncorrected distance and intermediate visual acuities, contrast sensitivity, incidence of photic phenomena, and patient satisfaction were not significantly different between the two groups (p > 0.05). However, the binocular uncorrected near visual acuities and spectacle independence rates were significantly better in the Mini-monovision group (p < 0.001). Conclusively, the pseudophakic mini-monovision technique using enhanced monofocal IOLs may be a promising option for presbyopia correction in patients unsuitable for multifocal IOLs.

## Introduction

Cataract is one of the main causes of visual impairment worldwide^1^, and cataract surgery using phacoemulsification and intraocular lens (IOL) implantation is one of the most commonly performed surgical procedures today^2^. As a result, the original goal of restoring blurred vision at far distance has evolved into the more complicated objective of improving vision at all functional distances with total spectacle independence^3^. The higher expectations and growing demands of patients have led to an advance in IOL designs^4^ and to the introduction of innovative combinations involving IOL implantation^5,6^.

Monofocal IOLs still remain the most frequently implanted IOLs today due to their relatively low cost, excellent outcomes for single-focus vision at far or near distance, and low incidence of photic phenomena such as halo and glare^7^. In patients with comorbidities such as corneal or macular diseases, for whom the use of multifocal IOLs is not recommended^8^, monofocal IOLs are good alternative candidates. Nevertheless, monofocal IOLs do not allow for complete spectacle independence for everyday activities^9^.

Multifocal IOLs are designed to split incident light into two or more points of focus (e.g., bifocal or trifocal), offering the advantage of improved vision for both far and near distances, which allows for spectacle independence^10^. However, the larger out-of-pocket expenses^7^, higher incidence of photic phenomena^11^, decreased contrast sensitivity^12^, restricted patient selection^8^, and the requirement of neural adaptation^13^ are imperative drawbacks to the implantation of multifocal IOLs. Even newer generation IOLs, developed to address these concerns^4^ (e.g., diffractive extended depth of focus [EDOF] IOLs), have been reported to cause undesired photic phenomena^14^.

The Tecnis Eyhance (ICB00) IOL (Johnson & Johnson Vision, Santa Ana, CA, USA) is a newly introduced enhanced monofocal IOL designed to provide improved intermediate vision while eliminating the disadvantages of multifocal IOLs. The refractive design has a modified anterior surface and continuous power profile from the periphery to the center of the lens, allowing for a wider range of vision without an increase in photic phenomena or a decrease in contrast sensitivity. However, its limitation in near vision correction^15^ is a major hindrance to the achievement of complete spectacle independence. As an alternative to new IOL designs, new bilateral implantation techniques, such as pseudophakic monovision^16^, have been applied to achieve a wider range of optimal vision.

Monovision is a surgical option in which the dominant eye is corrected for distance vision, whereas the non-dominant eye is corrected for near to mid-range vision^17^. The amount of intended residual myopia can be modified according to patient demands; the mini-monovision procedure aims for less residual myopia, ranging from anywhere between – 0.75 to – 1.75 diopters (D)^5^. Numerous studies have reported satisfactory spectacle independence following mini-monovision using monofocal IOLs^18–22^, with a few studies showing comparable results to those achieved by multifocal IOLs^20,23^.

Since its introduction, a few studies have compared the clinical outcomes of bilateral Eyhance IOL implantation to those of bilateral Tecnis 1-piece (ZCB00) IOL (Johnson & Johnson Vision, Santa Ana, CA, USA) implantation^24–27^. However, no studies to date have studied the clinical outcomes of pseudophakic mini-monovision using Eyhance IOLs.

This study aimed to evaluate the visual outcomes at far, intermediate, and near distances, refractive outcomes, defocus curves, contrast sensitivity, visual symptoms, spectacle independence, and satisfaction rates of patients who were bilaterally implanted with the new enhanced monofocal Eyhance IOLs using the mini-monovision technique to improve near vision. The clinical outcomes were compared to those of patients whose eyes were bilaterally targeted for far distance vision.

## Methods

### Study design and patients

This study was a retrospective, observational case series involving patients who underwent immediately sequential bilateral cataract surgery using the Eyhance IOL at a single institute between June 2020 and June 2021. This study was approved by the Institutional Review Board (IRB) of Yonsei University Health System (IRB Protocol Number 4-2021-0690). Waiver for informed consent was approved by the IRB. The study was conducted in accordance with the tenets outlined in the Declaration of Helsinki.

We retrospectively reviewed the clinical data from patients who had undergone same-day bilateral femtosecond laser-assisted cataract surgery (FLACS), performed by a single surgeon, at Severance Hospital in Seoul, South Korea. Patients were included if they were over 40 years of age and presented with a preoperative corneal astigmatism < 1.50 D in both eyes. Patients were excluded if they were over 85 years of age, had an axial length > 26.00 mm or < 22.00 mm, had previous ocular trauma or ocular surgery (including corneal and refractive surgery), or had any ocular diseases other than cataract.

### Preoperative examination and mini-monovision assessment

All patients underwent a comprehensive preoperative ophthalmologic examination comprising of monocular and binocular UDVA, CDVA, intraocular pressure, manifest refraction, keratometry, auto-refraction, slit-lamp biomicroscopy, dilated funduscopy, specular microscopy (EM-4000, Tomey GmbH, Nuremberg, Germany), and optical biometry (IOLMaster 700, Carl Zeiss Meditec AG, Jena, Germany). Patients were given the option to choose between bilateral emmetropic targets or mini-monovision based on personal preference and desire for presbyopia correction. For those who agreed to pseudophakic mini-monovision, ocular dominance was determined using the “hole-in-card” test. A single experienced surgeon selected the IOL power for each patient based on the predicted postoperative spherical equivalent refractions using Barrett II formulas. For emmetropic targets, the IOL power closest to emmetropia was selected. For mini-monovision, the dominant eye was corrected for emmetropia, and the non-dominant eye was targeted for a residual refraction of – 0.75 D.

### Intraocular lenses

The Tecnis Eyhance (ICB00) IOL is a single-piece, biconvex, acrylic hydrophobic foldable posterior chamber lens with a total diameter of 13.0 mm and an optic diameter of 6.0 mm. It has a spherical posterior surface and a modified aspheric anterior surface that are designed to achieve corrected distance vision and improved intermediate vision than that achieved by the standard aspheric monofocal IOL. Under slit lamp examination, this IOL is indistinguishable from Tecnis 1-piece monofocal (ZCB00) IOLs. A dioptric range from + 5.0 D to + 34.0 D, in 0.5 diopter increments, is available for use. The lenses’ optical A-constant is 119.3 and the spherical aberration is – 0.27 μm.

### Surgical technique

All operations were performed by a single experienced surgeon (T.K.), under topical anesthesia (proparacaine hydrochloride 0.5%). For FLACS, the LenSx platform (Alcon Laboratories, Inc., Fort Worth, TX, USA) was used to perform capsulorhexis and nucleus fragmentation. In eyes with astigmatism greater than 0.75 D, femtosecond laser-assisted arcuate keratotomy was performed based on the measurements of corneal astigmatism axis using the Verion corneal topography system (Alcon Laboratories, Inc.). The penetrating keratotomy incision was set at a corneal thickness depth of 80% and an arc diameter of 9.0 mm. A laser corneal incision was created at temporal, and corneal incision sites were carefully dissected using a Sinskey hook and the incised anterior capsule button was removed using forceps. The Centurion Vision System (Alcon Laboratories, Inc.) was used for conventional phacoemulsification, irrigation and aspiration, and polishing. The Eyhance IOL was implanted in the capsular bag; all incisions were closed with stromal hydration. A postoperative topical therapy of levofloxacin 1.5% eye drops, fluorometholone 0.1% eye drops, along with bromfenac sodium hydrate eye drops were instilled four times per day for 1 month.

### Postoperative outcome measures

The patients were examined 1 day, 1 week, 1 month, and 3 months postoperatively. In addition to the general postoperative ophthalmic examination, all patients underwent the following examinations at 3 months postoperatively:

###    Visual acuity

Distance visual acuities (UDVA at 4 m), intermediate visual acuities (UIVA at 66 cm), and near visual acuities (UNVA at 40 cm) were measured under photopic conditions (85 cd/m^2^) and 100% contrast with the Early Treatment Diabetic Retinopathy Study (ETDRS) charts and hand-held ETDRS vision cards (Precision Vision, Woodstock, IL, USA). All visual acuity measurements were converted into the logarithm of the minimum angle of resolution (logMAR) and Snellen equivalent values for statistical analysis.

###    Defocus curve

Binocular defocus curves were obtained with visual acuity measurements at 4 m of distance. Testing was conducted by consecutively adding lenses in gradual steps of – 0.50 D increments, from + 2.00 D to – 4.00 D of defocus.

###    Contrast sensitivity

Contrast sensitivity with best distance correction was determined under photopic (85 cd/m^2^) and mesopic (3 cd/m^2^) conditions using the Optec 6500 Vision Tester (Stereo Optical Company, Chicago, IL, USA). Sensitivity was determined at five different points of stimulus spatial frequencies ranging from 1.5 to 18 cycles per degree (1.5, 3, 6, 12, and 18).

###    Questionnaire

Patients were administered a questionnaire regarding discomfort in their daily lives due to postoperative photic phenomena (halo, glare, starburst) and spectacle dependence for everyday activities (at distance, intermediate, and near distances) 3 months after cataract surgery. “Do you experience discomfort in your daily life due to halo/glare/starburst? (Yes/No).” “Do you need spectacles to perform everyday activities at distance/intermediate/near? (Yes/No).” Patients were also asked about their satisfaction with the outcomes, and whether they would recommend the same operation to others. “Are you satisfied with the outcomes of cataract surgery using Eyhance intraocular lens? (Yes/No).” “Would you recommend cataract surgery using Eyhance intraocular lens to your friends or relatives? (Yes/No).” In addition, discomfort when walking on uneven surfaces or staircases, and incidence of fall injuries were investigated. A copy of the questionnaire is provided as Supplementary Information (Table S1).

### Statistical analyses

All statistical analyses were performed using SPSS version 18.0 (SPSS, Inc., Chicago, IL, USA). Pearson’s chi-square test was used for testing difference in frequencies. Student’s t-test was used to compare preoperative patient demographics and baseline values between the two groups. Preoperative and postoperative outcomes were compared between the two groups using the student’s t-test. P values < 0.05 were considered statistically significant. The number of patients who answered “yes” to each question on the patient questionnaire was calculated as a percentage.

## Results

### Baseline characteristics

Table 1 presents the demographic and preoperative ophthalmic measurements of all participants included in the Emmetropia and Mini-monovision groups. A total of 100 eyes of 50 patients who underwent bilateral cataract surgery using the Eyhance IOL were evaluated in this study. The mean age (± standard deviation) of the participants was 72.2 ± 5.43 in the Emmetropia group and 71.92 ± 9.98 in the Mini-monovision group (p = 0.90). The preoperative spherical equivalent (SE) was 0.54 ± 1.73 D in the Emmetropia group and 0.07 ± 2.61 in the Mini-monovision group (p = 0.30). The preoperative corneal astigmatism was 0.47 ± 0.34 D in the Emmetropia group and 0.50 ± 0.31 D in the Mini-monovision group (p = 0.76). The number of eyes requiring arcuate keratotomies did not differ between the two groups. The preoperative characteristics of the patients in the two groups did not differ significantly in terms of age, axial length, preoperative SE, and preoperative corrected distance visual acuity (CDVA; p > 0.05). All surgical procedures were uneventful and all IOLs were implanted into the capsular bag. No intra-operative or postoperative complications such as cystoid macular edema, endophthalmitis, secondary glaucoma, and posterior capsular opacification were noted. None of the patients underwent repositioning of the IOL. All of the patients included in the analysis completed 3 months of follow-up.

**Table 1:** Patient characteristics and ophthalmic measurements of patients bilaterally implanted with the Eyhance ICB00 intraocular lens.

	Emmetropia	Mini-monovision	p-value
Patients/eyes (n)	25/50	25/50	–
Male/female (n)	10/15	12/13	0.57
Age (years)	72.2 ± 5.43	71.92 ± 9.98	0.90
Monocular CDVA (LogMAR)	0.28 ± 0.31	0.25 ± 0.22	0.66
Binocular CDVA (LogMAR)	0.20 ± 0.25	0.15 ± 0.11	0.29
Axial length (mm)	23.79 ± 0.81	23.66 ± 1.19	0.51
Spherical equivalent (D)	0.54 ± 1.73	0.07 ± 2.61	0.30
Mean corneal keratometry (D)	44.18 ± 0.57	44.21 ± 0.56	0.87
Preoperative corneal astigmatism (D)	0.47 ± 0.34	0.50 ± 0.31	0.76
Arcuate keratotomy (n)	4	4	–

Data are expressed as mean ± standard deviation.

*CDVA* corrected distance visual acuity, *D* diopter, *logMAR* log of the minimum angle of resolution.

### Refractive and visual outcomes

**Figure 1:**
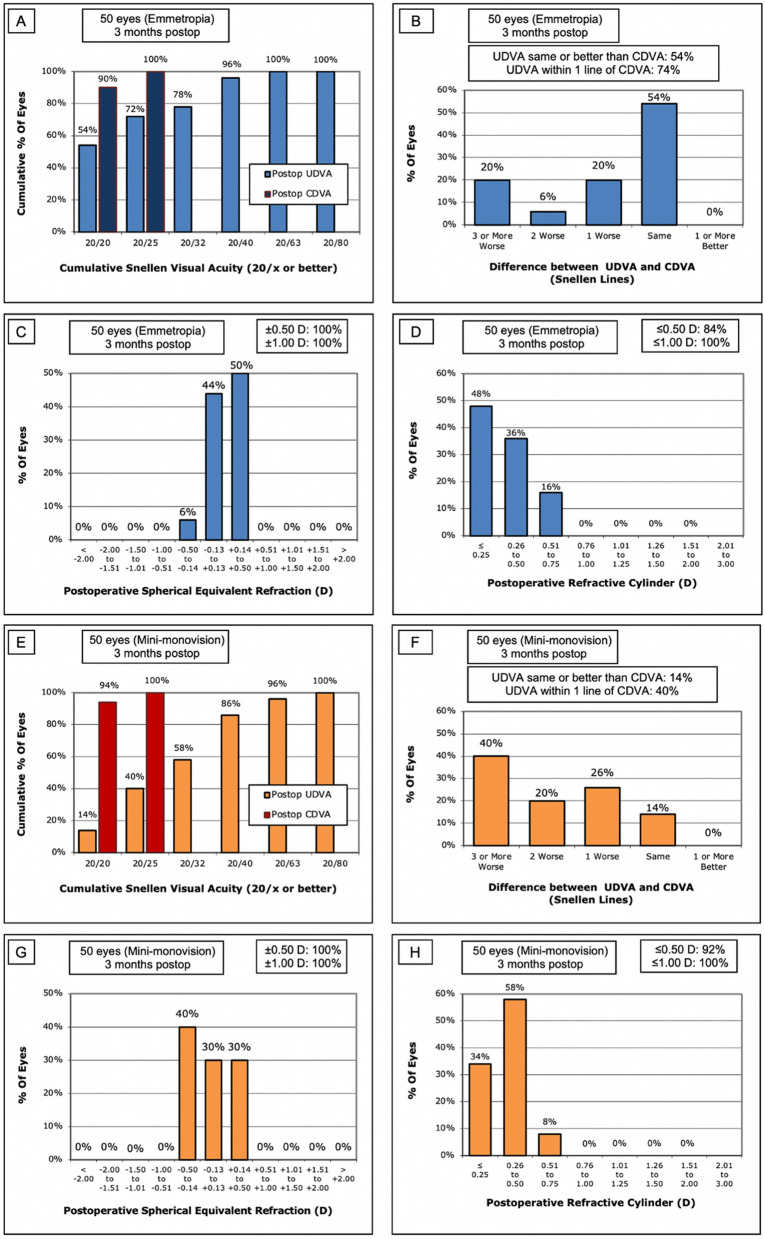
Standard graphs for reporting refractive outcomes for Emmetropia (**A**–**D**) and Mini-monovision (**E**–**H**) groups. (**A**, **E**) Cumulative percentage of postoperative UDVA and CDVA, (**B**, **F**) Cumulative percentage of lines of difference between postoperative UDVA and CDVA, (**C**, **G**) Postoperative spherical equivalent refraction relative to the intended target, (**D**, **H**) Postoperative refractive cylinder. *D* diopter.

Table 2 summarizes the postoperative refractive outcomes using modified refraction at 3 months after cataract surgery. The Emmetropia group had a mean postoperative SE of – 0.18 ± 0.21 D, whereas the Mini-monovision group had a mean postoperative SE of – 0.19 ± 0.18 D in the dominant eye and – 0.95 ± 0.19 D in the non-dominant eye. As mini-monovision was performed, the difference between the postoperative SE of the Emmetropia group and that of the dominant eye of the Mini-monovision group was not statistically significant (p = 0.84). However, the difference between the postoperative SE of – 0.18 ± 0.21 D in the Emmetropia group and that of – 0.95 ± 0.19 D in the non-dominant eye of the Mini-monovision group was statistically significant (p < 0.001*). In addition, as expected, the postoperative SEs of the dominant and non-dominant eyes in the Mini-monovision group were significantly different (– 0.19 ± 0.18 D vs – 0.95 ± 0.19 D, p < 0.001*). Figure 1 displays standard graphs for reporting refractive outcomes for both the Emmetropia (A–D) and Mini-monovision (E–H) groups. An accuracy evaluation of postoperative spherical equivalent relative to the intended target showed that out of a total of 100 eyes, all eyes (100%) were within 0.50 D of the intended target.

**Table 2:** Refractive and visual outcomes in patients bilaterally implanted with the Eyhance ICB00 intraocular lens at 3 months postoperatively.

	Emmetropia	Mini-monovision	p-value
**Postoperative corneal astigmatism (D)**	0.38 ± 0.24	0.40 ± 0.21	0.67
**Postoperative spherical equivalent refraction (D)**	–0.18 ± 0.21		–
Dominant eye	–	–0.19 ± 0.18	0.84
Non-dominant eye	–	–0.95 ± 0.19	< 0.01*
Monocular UDVA (4 m, logMAR)	0.08 ± 0.12	0.18 ± 0.14	< 0.01*
Monocular UIVA (66 cm, logMAR)	0.17 ± 0.11	0.14 ± 0.10	0.04
Monocular UNVA (40 cm, logMAR)	0.35 ± 0.14	0.12 ± 0.11	< 0.01*
Binocular UDVA (4 m, logMAR)	0.07 ± 0.11	0.10 ± 0.11	0.18
Binocular UIVA (66 cm, logMAR)	0.15 ± 0.09	0.12 ± 0.09	0.17
Binocular UNVA (40 cm, logMAR)	0.33 ± 0.13	0.06 ± 0.06	< 0.01*

Data are expressed as mean ± standard deviation.

*D* diopter, *logMAR* log of the minimum angle of resolution, *UDVA* uncorrected distance visual acuity, *UIVA* uncorrected intermediate visual acuity, *UNVA* uncorrected near visual acuity.

*Statistically significant values are marked with an asterisk (p < 0.05).

The visual outcomes of all participants are also reported in Table 2. Postoperative visual outcomes were evaluated both monocularly and binocularly. Both the Emmetropia and Mini-monovision groups reached high levels of binocular uncorrected distance visual acuity (UDVA; 0.07 ± 0.11 vs 0.10 ± 0.11, p = 0.18) and uncorrected intermediate visual acuity (UIVA; 0.15 ± 0.09 vs 0.12 ± 0.09, p = 0.17), with no statistically significant differences between the two groups. However, binocular uncorrected near visual acuity (UNVA; 0.33 ± 0.13 vs 0.06 ± 0.06, p ≤ 0.001*) was significantly greater in the Mini-monovision group than in the Emmetropia group.

### Defocus curves

**Figure 2:**
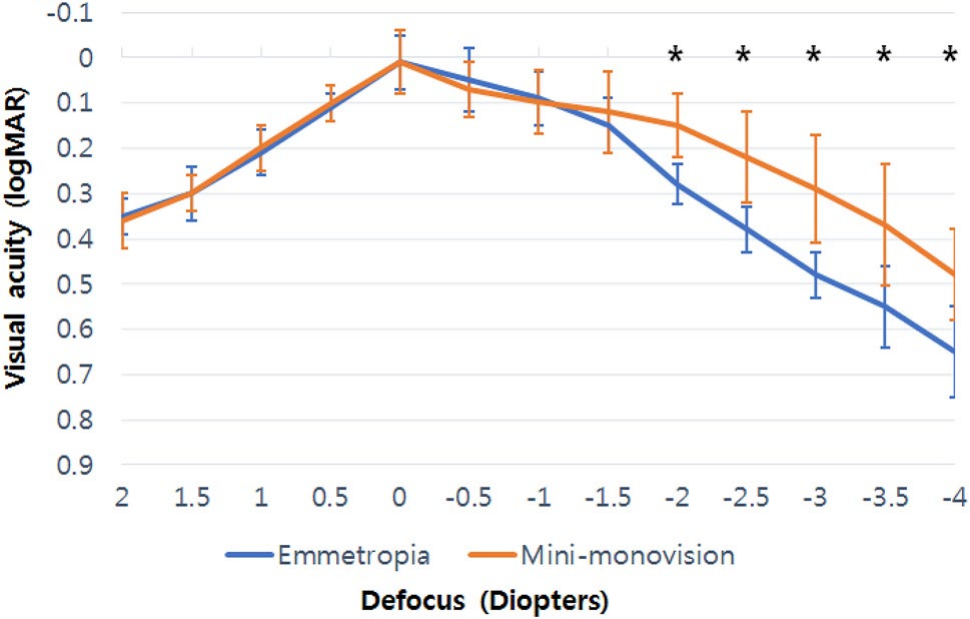
Mean binocular defocus curves of the Emmetropia and Mini-monovision groups using the Eyhance ICB00 intraocular lens. Vertical bars denote standard deviation. *logMAR* logarithm of the minimum angle of resolution.

The binocular defocus curves measured in the two groups are shown in Fig. 2. Both curves showed a peak corresponding to best visual acuity at 0.00 D (4 m) and reductions in visual acuity with gradual negative defocus. Both groups achieved a smooth and wide profile along the entire curve toward the myopic range, especially within the intermediate defocus range (near – 1.50 D defocus, corresponding to 66 cm). There were no statistically significant differences in the binocular visual acuity from a defocus between + 2.00 D and – 1.50 D. However, regarding the defocus range from – 2.00 D to – 4.00 D (corresponding to a reading distance range of 50–33 cm), the Mini-monovision group achieved significantly better binocular visual acuity and defocus results than the Emmetropia group (p < 0.05*).

### Contrast sensitivity

**Figure 3:**
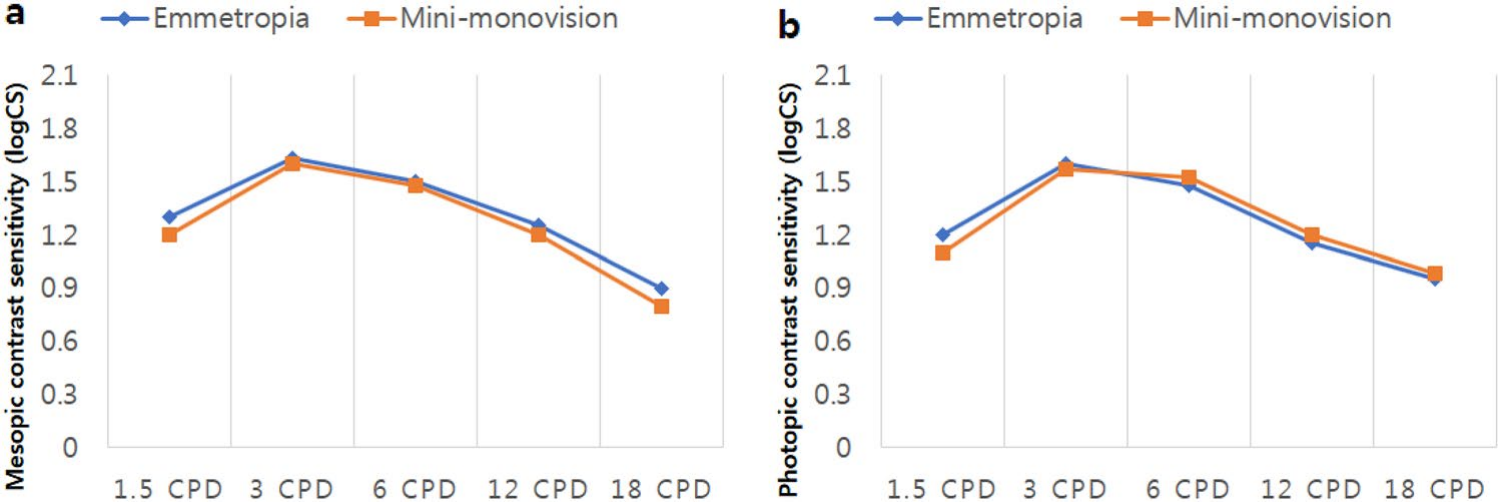
Contrast sensitivity test under photopic (**a**) and mesopic (**b**) conditions with implantation of the Eyhance ICB00 intraocular lens. *CPD* cycles per degree, *logCS* log contrast sensitivity.

Figure 3 shows the mean contrast sensitivity under both photopic and mesopic conditions with glare in the two groups. There were no statistically significant differences under low and high luminance conditions between the two groups for any spatial frequency (p > 0.05 for all comparisons).

### Patient questionnaire

Table 3 summarizes the findings of the patient questionnaire, which are presented as follows:

**Table 3:** Results of the patient questionnaire regarding visual symptoms, spectacle dependence, overall satisfaction, and recommendations in patients implanted with the Eyhance ICB00 intraocular lens.

	Emmetropia (n = 25)	Mini-monovision (n = 25)	All patients (n = 50)
**Photic phenomena**			
Halo	2 (8%)	2 (8%)	4 (8%)
Glare	0	0	0
Starburst	0	0	0
**Spectacle dependence**			
Distance	0	0	0
Intermediate	1 (4%)	1 (4%)	2 (4%)
Near	20 (80%)	5 (20%)	25 (50%)
**Overall satisfaction**	23 (92%)	24 (96%)	47 (94%)
**Recommendation**	25 (100%)	25 (100%)	50 (100%)

Data are expressed as frequency (percentage).

#### Visual symptoms

Two patients in the Emmetropia group and two patients in the Mini-monovision group reported discomfort due to halos. None of the patients in either group reported discomfort due to glare or starbursts. In total, four patients (8%) complained of any visual disturbances or photic phenomena.

#### Spectacle dependence

None of the patients in either group reported the need for far distance correction. One patient in the Emmetropia group and one patient in the Mini-monovision group reported the need for intermediate correction. However, 20 patients (80%) in the Emmetropia group reported the need for near vision correction in their everyday lives. On the contrary, only five patients (20%) in the Mini-monovision group reported the need for spectacles for near vision activities.

#### Overall satisfaction and recommendation

Overall, more than 90% of all patients from both groups were very satisfied with their optical outcomes. All patients in both groups (100%) reported that they would recommend the same type of IOL to others.

## Discussion

With the advent of digital technology that has occurred over the past decade, intermediate vision has reached paramount importance in our daily lives. Everyday activities, such as using computers and tablets, viewing the dashboard of a car, aisle shopping, applying makeup, cooking, and other activities performed at a standard arm length (60–70 cm) are considered to be within the intermediate vision range. Therefore, the changing needs and rising expectations of patients undergoing cataract surgery have led to the development of new IOL designs specifically targeting intermediate vision.

Since the introduction of the new enhanced monofocal Eyhance IOL, preliminary results of several studies have established its comparable refractive outcomes, good distance vision, superior intermediate vision, poor near vision with low spectacle independence, and similar rates of photic phenomena to those of the standard 1-piece ZCB00 IOL.

In agreement with previous studies^24–27^, the present study found excellent distance and intermediate visual outcomes in both the Emmetropia and Mini-monovision groups, as all participants underwent bilateral implantation of the Eyhance IOL. There were no statistically significant differences in the refractive error or the binocular UDVA and UIVA between groups, and the absolute visual outcome values in eyes that were bilaterally targeted for emmetropia were similar to those of available results^24–27^. However, the binocular UNVA was significantly better in the Mini-monovision group than in the Emmetropia group. The superior binocular UNVA outcomes of the Mini-monovision group are in accordance with previous findings from an earlier study conducted by Beiko^28^, in which mini-monovision using monofocal ZCB00 IOLs were compared to visual outcomes of accommodating IOLs.

The defocus curves of both groups further validated the visual outcome results. Predictably, both curves showed a peak corresponding to best visual acuity at 0.00 D (4 m) and reductions in visual acuity with gradual defocus. In accordance with the results of previous studies investigating Eyhance IOLs^27^, the Emmetropia group achieved a smooth and wide profile within the intermediate defocus range from – 0.50 D to – 2.00 D. The Mini-monovision group had comparable binocular defocus results in the distance and intermediate ranges compared to those of the Emmetropia group. However, our results showed that the Mini-monovision group presented with a longer plateau, and a sustained visual acuity that was better than that of the Emmetropia group within the near defocus range from – 2.00 D to – 4.00 D, which corresponds to reading distance. The pattern observed within the range from – 2.00 D to – 4.00 D appeared as a “rightward shift” of the Mini-monovision curve by nearly 0.50 D.

The lack of difference in binocular UIVA between the two groups is most likely due the small amount of anisometropia targeted in this study. As the non-dominant eye in the Mini-monovision group was targeted for a residual refraction of – 0.75 D, the resulting 0.75 D anisometropia was not significant enough to result in a statistically significant shift in the defocus curve. Accordingly, we were able to conclude that in the case of Mini-monovision using Eyhance IOLs targeted for 0.75 D anisometropia, there was no significant difference in binocular distance and intermediate vision, but a statistically significant difference in binocular near vision. Yet, further studies are needed to confirm whether statistically significant differences in UIVA is induced with higher anisometropia.

Understandably, contrast sensitivity was not significantly different between the two groups, as all participants were bilaterally implanted with the same IOL. The curve patterns are in concordance with those of previously published studies^27^. The observed absolute values were found to be similar to those previously reported and comparable to those of the monofocal 1-piece ZCB00 IOL.

Our most novel findings originate from the patient questionnaire. Expectedly, the rates of photic phenomena at 3 months postoperatively did not differ significantly between the two groups. Moreover, none of the patients complained of discomfort when walking on uneven surfaces or staircases and no cases of fall injuries were reported during the 3-month postoperative period. The low incidence of problems associated with stereopsis or neural adaptation should be noted in comparison to the high incidence associated with multifocal IOLs^13^.

In terms of spectacle dependence, less than 5% of patients in either group reported the need for correction for intermediate visual tasks. However, nearly 80% of patients in the Emmetropia group reported the need for near vision correction in everyday life, as opposed to only 20% of patients in the Mini-monovision group. Furthermore, the patients of the Mini-monovision group showed significantly greater spectacle independence at near distances; this qualitative finding is in accordance with the objective results exhibiting their superior UNVA and better defocus curves compared with those of the patients in the Emmetropia group.

Nevertheless, overall satisfaction and recommendation rates were found to be over 90% in both groups. This discrepancy from the objective findings is most likely due to the fact that both groups were informed, preoperatively, about the relative weakness of Eyhance IOLs in near vision correction. This disclosure may have been influential, as patients may have had low expectations for near vision improvements, leading to a high satisfaction rate in both groups.

A recent study conducted by Corbelli et al.^29^ compared the performance of the Eyhance IOL to that of the monofocal ZCB00 and EDOF IOLs (Tecnis Symfony ZXR00). The findings showed that the Eyhance IOLs achieved comparable intermediate vision to that achieved by the EDOF IOL^30^ and comparable dysphotopsia profiles to those achieved by a standard monofocal IOL. Considering the relative weakness in near vision correction presented by the Eyhance IOL, we aimed to provide additional insight by applying the mini-monovision technique to improve near vision. To our knowledge, this is the first study investigating pseudophakic monovision using bilateral Eyhance IOLs; as a result of this application, our preliminary findings report significantly better near visual acuity with better defocus curves and a significantly higher rate of spectacle independence in the Mini-monovision group than in the Emmetropia group.

This novel study is of paramount importance because its findings can broaden the range of patients that might benefit from this lens model in the future. The pseudophakic mini-monovision technique, employing the bilateral implantation of Eyhance IOLs, may serve as a solution for patients who demand more spectacle independence while avoiding the high costs and possible visual quality impairment associated with diffractive multifocal IOLs. In patients with ocular comorbidities, this approach may be a promising alternative option with unquestionable merit.

Nevertheless, the present study has some limitations, including the small sample size and the relatively short follow-up timeline. In addition, the retrospective nature of this study presents with potential confounding factors. Further prospective studies, including a larger number of patients, should compare near vision to trifocal IOLs to validate our findings. As IOL tilt or decentration may be an important variable negatively affecting the quality of vision, additional postoperative imaging should be performed in future prospective studies to check for tilt and decentration of the IOLs. In future studies, we also recommend the objective testing of two important parameters: reading speed and stereopsis. Finally, it would also be of interest to assess which refractive target provides the highest rates of satisfaction in patients undergoing the mini-monovision technique.

In conclusion, our findings showed good visual outcomes after the employment of the pseudophakic mini-monovision technique via the bilateral implantation of the Eyhance IOL, with similar performance for distance and intermediate vision but significantly better performance for near vision compared with those of bilateral emmetropic targets. For patients with retinal disorders or those with occupations that render them susceptible to dysphotopsias, pseudophakic mini-monovision with the enhanced monofocal IOL may be an ideal treatment choice for presbyopia correction.

## Supplementary Information

Supplementary Table S1.

## Data Availability

The datasets generated and analyzed during the current study are available from the corresponding author upon reasonable request.
