# Supplementary material for: Visual outcomes, spectacle independence, and patient satisfaction of pseudophakic mini-monovision using a new monofocal intraocular lens
Source: Sci Rep. 2022 Dec 15;12:21716. doi: 10.1038/s41598-022-26315-7 (PMC9755282; doi:10.1038/s41598-022-26315-7)
Supplement: Supplementary file 1 — Supplementary Table S1. [file 41598_2022_26315_MOESM1_ESM.docx]

**SUPPLEMENTARY MATERIAL**

**Table S1.** Patient questionnaire regarding visual symptoms, spectacle dependence and overall satisfaction.

| **Question** | **Answer** |
| --- | --- |
| 1. Do you experience discomfort in your daily life due to halo? | Yes / No |
| 2. Do you experience discomfort in your daily life due to glare? | Yes / No |
| 3. Do you experience discomfort in your daily life due to starburst? | Yes / No |
| 4. Do you need spectacles to perform everyday activities at distance vision?  (ex. TV) | Yes / No |
| 5. Do you need spectacles to perform everyday activities at intermediate vision?  (ex. Computer) | Yes / No |
| 6. Do you need spectacles to perform everyday activities at near vision?  (ex. Book) | Yes / No |
| 7. Are you satisfied with the outcomes of cataract surgery using Eyhance intraocular lens? | Yes / No |
| 8. Would you recommend cataract surgery using Eyhance intraocular lens to your friends or relatives? | Yes / No |


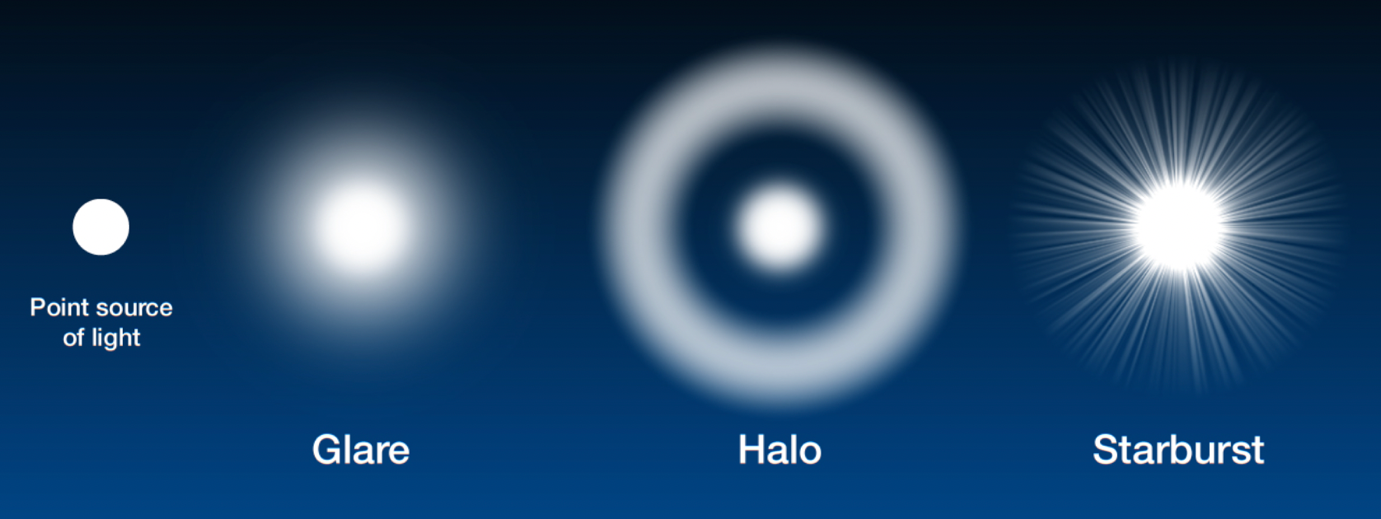


Image citation : Chang, Daniel. (2016). “Figure 1 : There are three distinct types of photopsias, or distortions of a point source of light.” Night Vision and Presbyopia-Correcting IOLs. Millennial Eye, Jul/Aug 2016. <https://millennialeye.com/articles/2016-jul-aug/night-vision-and-presbyopia-correcting-iols/>.
